# Migratory large vessel vasculitis preceding acute myeloid leukemia: a case report

**DOI:** 10.1186/s13256-017-1239-x

**Published:** 2017-03-16

**Authors:** Dinusha Chandratilleke, Anthea Anantharajah, Mauro Vicaretti, Warwick Benson, Lucinda J. Berglund

**Affiliations:** 1 Department of Immunopathology, Pathology West, ICPMR, Westmead Hospital, Sydney, 2145 NSW Australia; 2 Faculty of Medicine, University of Sydney, Sydney, NSW 2000 Australia; 3 Department of Vascular Surgery, Westmead Hospital, Sydney, 2145 NSW Australia; 4 Department of Haematology, Westmead Hospital, Sydney, 2145 NSW Australia

**Keywords:** Large vessel, Vasculitis, Acute myeloid leukemia, AML, Autoimmune, Myelodysplasia, MDS, Migratory

## Abstract

**Background:**

Large vessel vasculitis is a rare disorder usually occurring in the context of the autoimmune conditions of giant cell arteritis and Takayasu’s arteritis. Case reports have described large vessel vasculitis occurring in individuals with myelodysplastic syndrome, preceding transformation to acute myeloid leukemia.

**Case presentation:**

A 56-year-old Afghanistan-born woman presented with fever, a tender left carotid artery, and raised inflammatory markers. Computed tomography revealed thickening of the wall of her left carotid artery. Her symptoms resolved spontaneously; however, they recurred weeks later on the contralateral side, along with abdominal pain after eating. Further imaging with computed tomography and positron emission tomography demonstrated resolution of her left carotid artery abnormality, but new wall thickening and inflammation in her right carotid artery, abdominal aorta, and superior mesenteric artery. She was diagnosed as having large vessel vasculitis, which resolved with corticosteroids and methotrexate. Five months later, she developed acute myeloid leukemia. She had no known history of myelodysplastic syndrome at the time of diagnosis with vasculitis.

**Conclusions:**

Large vessel vasculitis in older individuals presenting with atypical clinical features, such as a migratory pattern of affected vessels, vessel wall tenderness, and marked systemic inflammation, should prompt a search for underlying myelodysplasia. Clinicians should be vigilant for progression to acute myeloid leukemia.

## Background

Large vessel vasculitis is a rare condition characterized by inflammation within the walls of the aorta and its major branches. It can occur in a range of autoimmune disorders, including Takayasu’s arteritis (TA) and giant cell arteritis (GCA), and typically requires treatment with high doses of corticosteroids and other immunosuppressive agents. In the literature, there have been case reports of large vessel vasculitis occurring in individuals with myelodysplastic syndrome (MDS), some of whom have later developed acute myeloid leukemia (AML). We describe a patient with an atypical presentation of large vessel vasculitis, which was migratory in nature and accompanied by marked systemic inflammatory features. Unlike previous case reports, our patient did not have a prior diagnosis of MDS, but subsequently developed AML.

## Case presentation

**Fig. 1:**
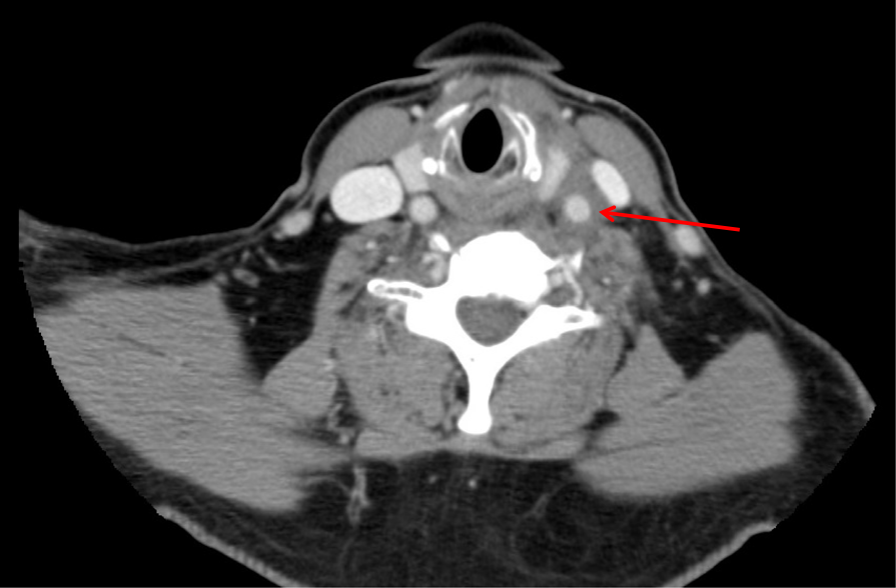
Computed tomography of the neck on initial presentation revealing wall thickening of the left distal common carotid artery with hazing of the adjacent fat (*red arrow*)

**Fig. 2:**
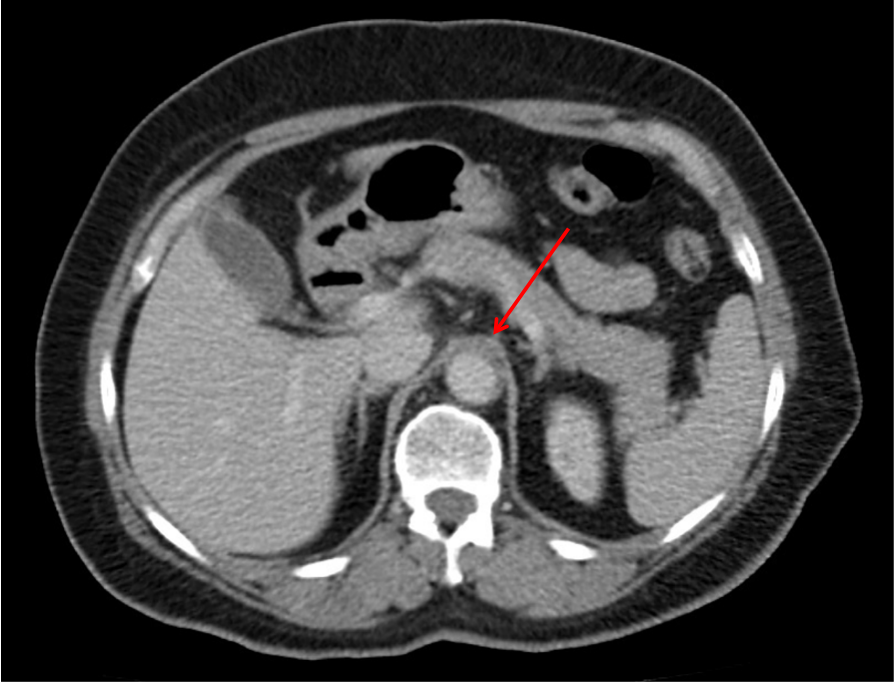
Computed tomography of the abdomen on second presentation revealing mild upper abdominal periaortic stranding with mural thickening of the aorta (*red arrow*), similar in appearance to the left common carotid artery from the original computed tomography of her neck (Fig. 1)

**Fig. 3:**
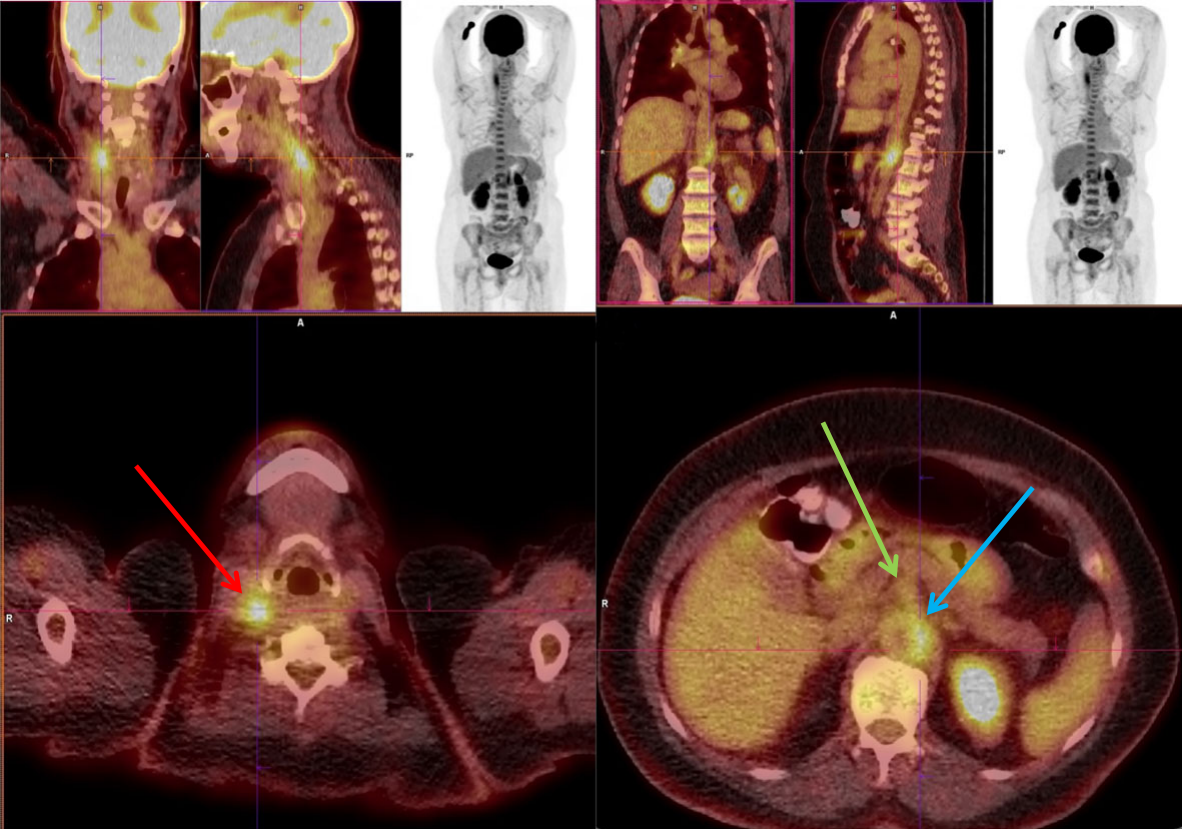
Positron emission tomography scan demonstrating diffuse, intense fluorodeoxyglucose uptake of the right common carotid artery (*red arrow*), abdominal aorta (*blue arrow*), and superior mesenteric artery (*green arrow*)

A 56-year-old Afghanistan-born woman presented to our hospital with a 2-week history of constant left-sided neck pain, associated fevers (38.0 °C), and rigors. Her past medical history included mild asthma and osteoarthritis. A physical examination revealed exquisite tenderness over the anterior triangle of her left neck. Laboratory studies showed a white cell count of 4.1 × 10^9^ cells/L (reference range 3.9 to 11.1), hemoglobin 112 g/L (115 to 165), mean corpuscular volume (MCV) 96 fL (82 to 98), C-reactive protein (CRP) 109 mg/L (≤3), with normal electrolytes and liver function tests. Computed tomography (CT) of her neck (Fig. 1) revealed wall thickening of her left distal common carotid artery with hazing of the adjacent fat. Autoimmune serology, including anti-nuclear antibodies, anti-neutrophil cytoplasmic antibodies, antibodies to extractable nuclear antigens, double stranded-deoxyribonucleic acid (DNA) antibodies, rheumatoid factor, and antibodies to cyclic citrullinated peptide, were negative. These results were helpful in excluding a systemic small vessel vasculitis. Her serum complement levels were normal, and a left temporal artery biopsy was negative. Her neck pain and fevers resolved spontaneously without corticosteroids or other immunosuppression and she was discharged home on day 6. She presented 17 days later with fever (38.0 °C) and abdominal pain 20 to 30 minutes after eating. A CT of her abdomen (Fig. 2) revealed mild upper abdominal periaortic stranding with mural thickening, similar in appearance to her left common carotid artery from the original CT scan of her neck. During the next 24 to 48 hours, she developed new right-sided neck pain, contralateral to her pain on initial presentation. A CT angiogram revealed improvement in her left common carotid artery thickening, interval development of new right common carotid artery thickening, and hazing around her upper abdominal aorta, coeliac axis, and superior mesenteric arteries. A positron emission tomography (PET) scan (Fig. 3) demonstrated diffuse, intense fluorodeoxyglucose (FDG) uptake of her right common carotid artery, abdominal aorta, and superior mesenteric artery, consistent with a large vessel vasculitis, which had presented with a systemic inflammatory response, carotidynia, and mesenteric angina.

She was treated with methylprednisolone (500 mg daily for 3 days) administered intravenously with immediate resolution of her abdominal and neck pain and fevers. She was subsequently commenced on methotrexate 15 mg/week administered orally and prednisone 50 mg/day. She remained well over the following 5 months, with continuation of methotrexate, and her prednisone dose was gradually reduced to 15 mg/day.

**Fig. 4:**
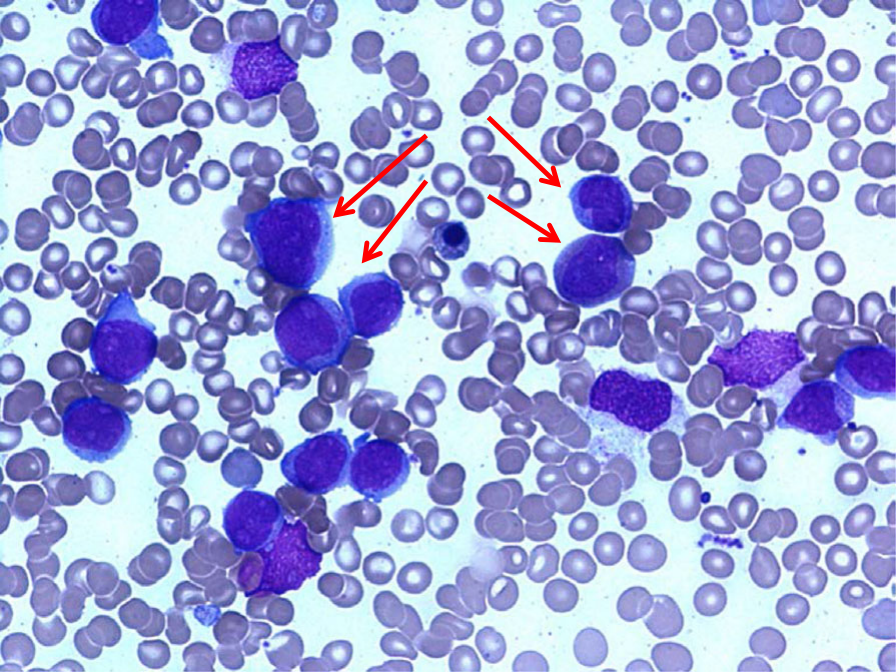
Bone marrow aspirate demonstrating features of acute myeloid leukemia including myeloblasts (*red arrows*) >20 %

Five months later, she reported fatigue and persistent cough despite multiple courses of antibiotics. Blood tests revealed neutropenia with >20 % blasts on peripheral blood film. A subsequent bone marrow aspirate (Fig. 4) demonstrated a markedly hypercellular marrow with 69 % blasts, which by flow cytometry were identified as an abnormal population of myeloid cells expressing CD117, CD13, CD33, human leukocyte antigen-antigen D related (HLA-DR), CD123, lacking CD34, with aberrant CD4 expression, consistent with AML. Molecular studies showed a FLT3-internal tandem duplication (ITD) mutation, which characterizes more aggressive disease and frequent relapses [1]. She received induction chemotherapy with cytarabine and idarubicin but a month later a bone marrow aspirate demonstrated residual blasts (9 %) consistent with persistent AML. She received re-induction chemotherapy with cytarabine and idarubicin, and then further re-induction with fludarabine, cytarabine, and idarubicin. Despite initial morphological remission, her AML relapsed 3 months later. She had many infective complications, including febrile neutropenia and *Clostridium difficile* colitis, but her large vessel vasculitis did not recur. Due to a rapidly rising blast count and refractory disease, she was palliated and died 8 months after her diagnosis of AML.

## Discussion

We present a case of large vessel vasculitis with atypical features, including a migratory involvement of affected vessels and marked systemic inflammatory features, with subsequent progression to AML.

Large vessel vasculitis associated with MDS has rarely been described [2]. In some cases, the diagnoses of MDS and large vessel vasculitis are made simultaneously, and in others, the MDS has been pre-existing. Our patient did not have a previous diagnosis of MDS before the development of vasculitis, and on presentation, had only minimally reduced hemoglobin (112 g/L). Over the following weeks, her peripheral blood neutrophil count fluctuated between normal and mildly reduced (1.4 to 3.4 × 10^9^ cells/L), as did her monocyte count (0.5 to 1.8 × 10^9^ cells/L); however, her platelet count remained normal. With high dose glucocorticoids, her neutrophil count rose to 6.0 × 10^9^ cells/L; however, the absence of a steroid-induced neutrophilia may also suggest a degree of myelodysplasia. The mild monocytosis, although not specific in the context of inflammation, may have been significant, given that her subsequent AML blasts aberrantly expressed CD4, which is normally expressed on monocytes.

Large vessel vasculitis can occur in autoimmune disorders, including extracranial GCA and TA. Marked systemic inflammation may be a feature of TA, however this disorder classically affects individuals younger than 50 years of age [3, 4]. GCA, more frequently seen in older individuals, typically involves smaller vessels such as the temporal arteries, but can also affect the aorta and its major branches. The few reported cases of MDS-associated large vessel vasculitis presented in a similar manner to our patient: at an age >50 years, with acute inflammation, including tender vessels and a highly raised CRP [2, 5, 6], exceeding the median CRP of 52 mg/L in patients with temporal artery biopsy-proven GCA [7].

In one review, five of eight patients with MDS-associated large vessel vasculitis developed AML, often refractory, usually within a year of presentation with vasculitis [2]. The timing of large vessel vasculitis preceding AML, and the unusual clinical features of acute inflammation with vessel wall tenderness and highly elevated inflammatory markers, suggest that the vasculitis in these patients may be a paraneoplastic phenomenon. The cytopenias in some forms of MDS may be immune mediated [8], with activated T cells inducing cytokine-mediated apoptosis of myeloid stem cells via tumor necrosis factor (TNF) and interferon gamma [5, 9]. Dysregulated immune mechanisms may thus be involved in the pathogenesis of MDS [10]. The association between MDS and autoimmune phenomena, such as arthritis, vasculitis, and connective tissue diseases, is well recognized in the literature [11, 12]; however MDS-associated vasculitis more commonly affects small caliber vessels [11, 13, 14].

Although the vasculitis in our patient responded promptly to glucocorticoids, the subsequent emergence of AML raises the possibility that immunosuppression impaired the cytotoxic anti-tumor response, and thus unmasked the AML. The majority of cases of patients reported with both large vessel vasculitis and MDS, however, received no steroid-sparing agent, yet several still progressed to AML [2]. In light of the poor prognosis when given corticosteroids, it is important to recognize the high risk of progression to AML in these individuals. Consideration may be given to simultaneous treatment for MDS, including azacitidine or even high intensity chemotherapy followed by allogeneic stem cell transplantation in selected patients with good performance status. A recent multicenter study confirmed that treatment with azacitidine in patients with both MDS and a systemic inflammatory or autoimmune disease appeared to have a positive effect on their inflammatory condition [15]; however, whether this treatment would reduce progression to AML in individuals with concurrent large vessel vasculitis is undetermined.

## Conclusions

In summary, we present a 56-year-old woman with a large vessel vasculitis with migratory features, vessel wall tenderness, and marked systemic inflammation, who subsequently developed AML. Large vessel vasculitis with atypical clinical features in older individuals should prompt a search for underlying myelodysplasia, and vigilance for progression to AML.

## Abbreviations

AML: Acute myeloid leukemia
CRP: C-reactive protein
CT: Computed tomography
DNA: deoxyribonucleic acid
FDG: Fluorodeoxyglucose
GCA: Giant cell arteritis
HLA-DR: Human leukocyte antigen-antigen D related
ITD: Internal tandem duplication
MCV: Mean corpuscular volume
MDS: Myelodysplastic syndrome
PET: Positron emission tomography
TA: Takayasu’s arteritis
TNF: Tumor necrosis factor
